# Impact of Low Skeletal Muscle Mass and Obesity on Hearing Loss in Asymptomatic Individuals: A Population-Based Study

**DOI:** 10.3390/healthcare10102022

**Published:** 2022-10-13

**Authors:** Chul-Hyun Park, Kyung Jae Yoon, Yong-Taek Lee, Sung Min Jin, Sang Hyuk Lee, Tae Hwan Kim

**Affiliations:** 1 Department of Physical and Rehabilitation Medicine, Kangbuk Samsung Hospital, Sungkyunkwan University School of Medicine, Seoul 03181, Korea; 2 Department of Otorhinolaryngology-Head and Neck Surgery, Kangbuk Samsung Hospital, Sungkyunkwan University School of Medicine, Seoul 03181, Korea; 3 Department of Otorhinolaryngology-Head and Neck Surgery, Samsung Medical Center, Sungkyunkwan University School of Medicine, Seoul 06351, Korea

**Keywords:** hearing loss, low muscle mass, pre-sarcopenia, obesity, pre-sarcopenic obesity

## Abstract

The relationship between low muscle mass (LMM) with obesity and hearing loss has been poorly studied. We aimed to investigate the association of LMM and obesity on hearing loss in the general population. A total of 265,792 adults who underwent a hearing test and body composition analyses were included. Pre-sarcopenia was defined as having an appendicular muscle mass index <5.7 kg/m^2^ for women and <7.0 kg/m^2^ for men, and obesity as a body mass index ≥25 kg/m^2^, while pre-sarcopenic obesity was defined as the co-presence of LMM and obesity. Participants were divided into four groups according to the presence of pre-sarcopenia and/or obesity. The prevalence of hearing loss was 1.8% in the control, 2.5% in the pre-sarcopenia alone, 3.0% in the obesity alone, and 6.2% in the pre-sarcopenic obesity group (*p* < 0.001). Hearing Thresholds were the highest in the pre-sarcopenic obesity group compared with the other three groups. In multivariable-adjusted models, the risk of hearing loss was the highest in the pre-sarcopenic obesity group (odds ratio: 1.30 [95% confidence interval: 1.10–1.56]), followed by the obesity alone (1.20 [1.12–1.28]) and pre-sarcopenia alone (1.19 [1.06–1.34]) group compared with the control group (*p* < 0.001). Pre-sarcopenic obesity was independently associated with a higher prevalence of hearing loss, supporting pre-sarcopenic obesity itself as a risk for the decline in hearing function.

## 1. Introduction

The prevalence of hearing loss (HL), a widespread public health issue, ranges from 4.9 to 17.0% [1], rises with advancing age, and is more common in men than women [2,3]. Hearing loss in adults is associated with communication difficulties and negative effects on an individual’s psychosocial function and cognition. It can bring social isolation and a low health-related quality of life [4,5,6]. Genetic factors, vascular causes, infections, and ototoxic medications are well-known risk factors for HL [4,5,7]. Recently, studies have also reported an association between HL and various metabolic problems, including diabetes mellitus (DM) [8], cardiovascular disease [9,10], and excess weight [11].

Pre-sarcopenic obesity is a condition where low skeletal muscle mass (called pre-sarcopenia) and obesity coexist, presenting as both increased adiposity with decreased muscle mass and function [12]. Several studies have reported that pre-sarcopenia is associated with metabolic disturbances such as insulin resistance, dyslipidemia, and atherosclerosis [13,14,15]. Some studies reported that in the condition of the co-existence of pre-sarcopenia and obesity, the risk of metabolic impairment and physical disability is higher than either pre-sarcopenia or obesity alone [16,17]. Furthermore, low muscle mass (pre-sarcopenia) itself is associated with coronary atherosclerosis, metabolic syndrome, and DM occurrence [15,18,19]. Because low muscle mass and obesity have similar pathophysiologic mechanisms, including inflammation, dietary factors, and physical activities, the coexistence of low muscle mass and obesity could increase the risk of mortality and morbidities and could result in more metabolic-related disorders. These several metabolic impairments could result in a decrease in cochlear function, and thus it could be assumed that HL and pre-sarcopenia are also related.

At present, only two studies have reported that pre-sarcopenia is associated with the prevalence of HL [20,21]. However, no study has investigated the impact of the coexistence of low muscle mass and obesity (pre-sarcopenic obesity) on HL. Given the fact that both pre-sarcopenic obesity and HL are associated with metabolic disturbances, we assume that the condition may be intrinsically related to the prevalence of HL. Therefore, we stratified a cohort of adults by the presence of pre-sarcopenia and/or obesity and assessed their risk for hearing loss, which was evaluated by pure-tone audiometry (PTA) testing.

## 2. Methods

### 2.1. Participants

This cross-sectional, two-center, retrospective cohort study was a component of the Kangbuk Samsung Health Study. The subjects were participants in a medical regular health check-up program in the two Kangbuk Samsung Hospital Total Health Centers, Sungkyunkwan University in Suwon and Seoul, South Korea [22]. The aim of this medical health check-up program was to monitor the health of employees in Korea via regularly screening to detect a disease. The subjects of this study consisted of a subset of participants who took a PTA test and were included in a bioelectrical impedance analysis from January 2012 to December 2017 (*n* = 290,329).

**Figure 1:**
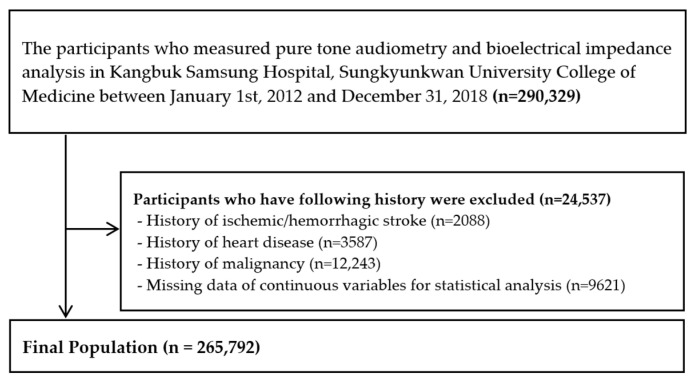
Selection of study participants.

For this study, we excluded 24,537 participants who met the following exclusion criteria: history of ischemic/hemorrhagic stroke (*n* = 2088), history of heart disease (*n* = 3587), history of malignancy (*n* = 12,243), and missing data for laboratory parameters (*n* = 9621) (Figure 1). Some subjects met more than one exclusion criteria, leaving 265,792 subjects enrolled in this final analysis.

This protocol was carried out according to the Declaration of Helsinki and it was approved by the Institutional Review Board of Kangbuk Samsung Hospital (KBSMC no. 2022-03-036), which waived the requirement for written informed consent because of the anonymized data obtained as part of regular medical examinations.

### 2.2. Measurements

The participants were examined after a 12 h overnight fasting. Blood samples were collected to estimate levels of glycated hemoglobin (HbA1c), fasting glucose, triglycerides, total cholesterol, low-density lipoprotein cholesterol (LDL-C), high-density lipoprotein cholesterol (HDL-C), aspartate transaminase (AST), and alanine aminotransferase (ALT). Fasting blood glucose levels (mmol/L) were evaluated with a nephelometric assay using a BNII nephelometer (Deerfield, Dade Behring, IL, USA). HbA1c was estimated with an immune-turbidimetric assay using a Cobra Integra 800 analyzer (Roche Diagnostics, Switzerland). Lipid profiles were estimated with an enzymatic colorimetric assay. The hexokinase method was used to calculate the glucose levels of the blood. A calorimetric test was used to measure triglycerides and total cholesterol levels. The enzymatic calorimetric test was used to measure LDL-C. The selective inhibition method was used to estimate HDL-C.

Data on the participants’ smoking status, alcohol history, and medical history of DM and hypertension (HTN), heart disease, dyslipidemia, ischemic/ hemorrhagic stroke, and cancer were collected by trained physicians using standardized, self-administered questionnaires. A history of hypertension (HTN) was concluded if the participant’s blood pressure ≥140/90 mmHg or if they were presently taking antihypertensive medication according to the criteria outlined in the 8th report of the Joint National Committee on the prevention, detection, evaluation, and treatment of high blood pressure [23]. The history of DM was defined using the criteria of the American Diabetes Association and the subjects’ answer to the questionnaire. Smoking status was categorized into never, former, and current smoking categories. Participants with alcohol consumption >20 g/day were grouped into a heavy drinking group. Physical activities were assessed using the International Physical Activity Questionnaire-Short Form [24]. Participants who performed vigorous exercise over 3 times a week for over 20 min/session were grouped into a regular physical activity group.

Anthropometric data were measured by experienced and trained nurses. Each participant’s weight and height were measured twice and the two measurements were averaged. Their body mass index (BMI) was calculated as their body weight in kilograms (kg) divided by their height in meters squared (kg/m^2^). Appendicular skeletal muscle mass (kg), the sum of the muscle mass of the legs and arms, was calculated with a bioelectrical impedance analysis (BIA) using eight-point tactile electrodes (InBody 720, Biospace, South Korea). The BIA was calibrated every day prior to the test and was validated for accuracy and reproducibility for estimating skeletal muscle mass.

### 2.3. Measurement of Audiometric Measurements

Pure-tone audiometric (PTA) testing was conducted by experienced audiometric technicians using a GSI 67 audiometer (Bedford, USA) equipped with TDH-39 supra-aural earphones (TelephonicsCo., Farmingdale, NY, USA) in an equipped sound-attenuating booth. Air conduction levels were measured in dB for both ears at 0.5 kHz, 1.0 kHZ, and 2.0 kHz. The presence of HL was defined as an average of pure-tone air thresholds at 0.5 kHz, 1.0 kHz, and 2.0 kHz > 25 dB in both right and left ears. Low-frequency and mid-frequency values were categorized by calculating the pure tone averages at 0.5 kHz, 1 kHz, and 2 kHz. For quality control, the audiometer was annually calibrated according to the standards of the American National Standards Institute (ISO 389:1991), and biological calibrations were performed daily prior to the first examination. The ambient noise was checked with a sound level meter.

### 2.4. Definition of Pre-Sarcopenic Obesity

To define the term pre-sarcopenia, the appendicular skeletal muscle mass index (SMI) was measured as the ratio of the appendicular skeletal muscle mass and height square (kg/m^2^). The low skeletal muscle mass, pre-sarcopenic status was defined by the criteria of the Asian Working Group for pre-sarcopenia (AWGS) (SMI of below 7.0 kg/m^2^ in men and below 5.7 kg/m^2^ in women) [25]. Obesity was defined as a BMI ≥ 25.0 (kg/m^2^). Pre-sarcopenic obesity was defined as the co-presence of obesity and pre-sarcopenia [26]. The subjects were grouped into four categories as follows: control (without pre-sarcopenia and obesity), pre-sarcopenia alone, obesity alone, and pre-sarcopenic obesity.

### 2.5. Statistical Analysis

The baseline characteristics of the groups were assessed with a Chi-square test for categorical variables and a one-way analysis of variance (ANOVA) for the continuous variables. To evaluate the association of pre-sarcopenic obesity with the prevalence of hearing loss, a binary logistic regression model was used to calculate odds ratios (ORs) with 95% confidence intervals (CIs) for hearing loss as a dependent variable. ORs were calculated as risks for the presence of hearing loss in the pre-sarcopenia alone group, the obesity alone group, and the pre-sarcopenic obesity group compared to the control group. We used three models to progressively adjust for confounding factors. Model 1 was the crude analysis without adjustments. Model 2 was adjusted for sex, age, screening center, current smoker, heavy drinker, and regular physical activity. In Model 3, we additionally adjusted for HTN, HbA1c, and HDL-C. Stratified analyses for subgroups divided by sex (women vs. men) and age (≥60 vs. <60 years) were performed. The statistical significance was set at *p* < 0.05. All analyses were performed using IBM SPSS version 23.0 (IBM Co., New York, NY, USA).

## 3. Results

### 3.1. Baseline Characteristics

Of the 265,792 total participants, there were 145,240 (54.6%) men and 120,552 (45.4%) women. Their mean (±SD) age and BMI were 42.3 ± 9.4 years and 23.8 ± 3.5 kg/m^2^, respectively (Table 1). The participants were divided into four groups according to the presence or absence of pre-sarcopenia and/or obesity as follows: (1) subjects without either abnormality as a control group (*n* = 153,809; 57.9%); (2) subjects with pre-sarcopenia alone (*n* = 25,981, 9.8%); (3) subjects with obesity alone (*n* = 80,923, 30.4%); and (4) subjects with both abnormalities or pre-sarcopenic obesity (*n* = 5079, 1.9%) (Table 2). Differences in the demographic characteristics among the four groups were significant for all variables (all *p* < 0.0001). The mean SMIs were 7.3 (1.0) in the control, 5.6 (0.5) in the pre-sarcopenia alone, 7.8 (0.9) in the obesity alone, and 5.9 (0.6) in the pre-sarcopenic obesity group. The mean BMIs were 22.7 (2.3) in the control, 19.2 (1.4) in the pre-sarcopenia alone, 27.2 (3.0) in the obesity alone, and 5.9 (0.6) in pre-sarcopenic obesity group.

### 3.2. Prevalence of Hearing Loss and Hearing Threshold in Pre-Sarcopenia, Obesity, Pre-Sarcopenia Obesity Groups

Among the total population, 5837 (2.2%) participants had hearing loss. The prevalence of hearing loss was 1.7% in the control group, 2.2% in the pre-sarcopenia alone group, 2.9% in the obesity alone group, and 6.2% in the pre-sarcopenic obesity group, with the pre-sarcopenic obesity showing the highest prevalence (*p* < 0.001). 

**Figure 2:**
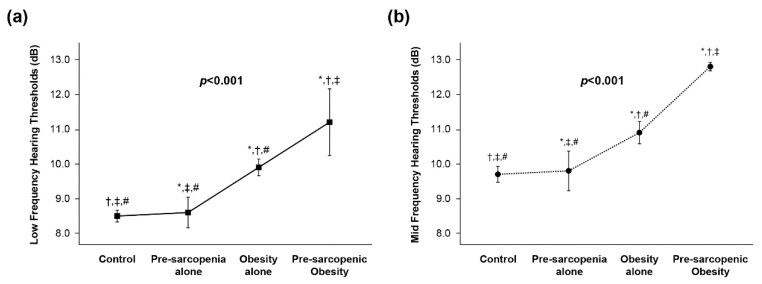
Comparison of (**a**) low-frequency Hearing Thresholds and (**b**) mid-frequency Hearing Thresholds between study groups. Adjusted means of hearing threshold in the study group were estimated from ANCOVA after adjustments for age, sex, screening center, heavy alcohol, smoking status, history of hypertension, HbA1c, and HDL-C. * Adjusted *p* < 0.001 versus control group in post hoc analysis. ^†^ Adjusted *p* < 0.001 versus pre-sarcopenia alone group in post hoc analysis. ^‡^ Adjusted *p* < 0.001 versus obesity alone group in post hoc analysis. # Adjusted *p* < 0.001 versus pre-sarcopenic obesity group in post hoc analysis. Abbreviations: HbA1c, glycated hemoglobin; HDL-C, high-density lipoprotein cholesterol.

A comparison of low-frequency and mid-frequency Hearing Thresholds between the study groups are presented in Figure 2. The low-frequency Hearing Thresholds (dB) in the control, pre-sarcopenia only, obesity only, and pre-sarcopenic obesity group were 8.5, 8.6, 9.9, and 11.2, respectively (*p* < 0.001) (Figure 2a). The mid-frequency hearing thresholds in the control, pre-sarcopenia alone, obesity alone, and pre-sarcopenic obesity group were 9.7, 9.8, 10.9, and 12.8, respectively (*p* < 0.001) (Figure 2b).

### 3.3. Association between Hearing Loss and Pre-Sarcopenic Obesity

When the univariant logistic regression analysis was performed, the OR increased progressively toward pre-sarcopenia alone, obesity, and pre-sarcopenia obesity (1.27 (1.15–1.39), 1.70 (1.61–1.80), and 3.77 (3.34–4.25), respectively (Table 3, *p* value < 0.0001). After adjustments for possible confounding factors, including age, sex, screening center, heavy alcohol, smoking status, and regular physical activity in Model 2, the OR was attenuated, but statistical significance was still observed. When hypertension, HbA1c, and HDL-C were added to Model 2 to identify the effects of metabolic factors, a similar statistical significance was also observed in Model 3 (adjusted OR, CI): 1.19 (1.06–1.34), 1.20 (1.12–1.28), and 1.30 (1.10–1.56) in pre-sarcopenia alone, obesity, and pre-sarcopenia obesity, respectively (*p* value < 0.0001).

### 3.4. Subgroup Analyses by Sex and Age

The association between pre-sarcopenic obesity and the prevalence of hearing loss was next examined for sex and age subgroups (Table 4). The association between pre-sarcopenic obesity and hearing loss was significant only in women (adjusted OR: 1.44; 95% CI: 1.16–1.80), not in men (adjusted OR: 1.15; 95% CI: 0.90–1.47). Furthermore, younger participants (<60 years) with pre-sarcopenic obesity had an increased risk of hearing loss (adjusted OR: 1.43; 95% CI: 1.11–1.84), which was not significant in older participants (≥60 years) (adjusted OR: 1.18; 95% CI: 0.95–1.46).

## 4. Discussion

In this study, we observed that pre-sarcopenic obesity was independently associated with an increased risk of hearing loss in a population-based study. These associations remained significant even after adjustments for possible confounding variables. Furthermore, we found a considerably strong association between pre-sarcopenic obesity and hearing loss in younger (<60 years) and women participants. This is the first study to report pre-sarcopenic obesity as a risk factor for hearing loss, measured by PTA testing, with sex and age differences in a healthy adult population. 

There are only two previous studies that showed the association between sarcopenia and HL. Kang et al. [20] reported an association between sarcopenia and hearing thresholds in postmenopausal women. The adjusted OR for mild HL was 1.58 (95% CI, 1.131–2.217; *p* = 0.007) and the adjusted OR for profound HL was 2.667 (95% CI, 1.866–3.812; *p* < 0.001) in the sarcopenia group relative to the normal group. Lee et al. [21] reported a relationship between sarcopenia and HL in older Koreans aged 60 years or old. They categorized appendicular muscle mass in tertiles. The OR (95% CI) of hearing loss was 1.57 (0.92–2.68) in the middle tertile and 1.79 (1.03–3.08) in the lowest tertile, compared with the highest tertile in the female population. The male population did not show statistical significance between hearing loss and muscle mass. The result that the female group showed a stronger association with hearing loss is consistent with our study. From what we can see, no other study has been conducted to show an association of the co-existence of low muscle mass with obesity on hearing loss spanning the entire range of age.

Sarcopenia has been reported as a risk factor for metabolic syndrome, limiting physical and daily-life activities. In addition, sarcopenia could result in arterial stiffness and hypertension [27,28,29,30]. One study reported that when sarcopenia coexists with obesity, so-called pre-sarcopenic obesity, the group had a higher risk of metabolic syndrome than the sarcopenic group (OR: 1.98, 95% CI: 1.25–3.16) and the obesity group (OR: 7.53, 95% CI: 4.01–14.14) [31]. Several studies reported that hearing loss is associated with metabolic syndrome [32,33] including a systemic review and meta-analysis [34]. Therefore, there may be an association between pre-sarcopenia and HL, and it can be assumed that the association with sarcopenic obesity and HL is higher than that of sarcopenia only. As can be seen from our study, both the prevalence of HL and the risk of hearing loss (odds ratio for the prevalence of HL) gradually increased from normal in the pre-sarcopenia alone, obesity alone, and pre-sarcopenic obesity group.

HL is associated with cardiovascular disease [9]. The stria vascularis in the cochlear is highly vascularized and sensitive to the alteration of the arterial blood supply [35]. Several studies reported that changes in blood flow to the cochlear affected cochlear function both in animal models [36,37] and also in humans [10]. The association between pre-sarcopenic obesity and cardiovascular disease has been well known [38]. Greater muscle mass requires more blood flow, and this leads to a higher cardiac output and size adaptation of the arteries. Consequently, patients may be more likely to have greater arterial stiffness in small diameter arteries. This may result in the reduction in cochlear blood flow and decreased blood supply to the cochlear, which can lead to hearing impairment. Conversely, an increased muscle mass may reduce blood pressure and improve hemodynamics [39], suggesting that muscle mass could play a protective role in the development of cardiovascular disease and thus could preventing hearing impairment. The interesting result is that the female population has a stronger association with cardiovascular disease than the male population [9]. Moreover, poor blood flow by arterial stiffness was more significantly associated with females than males [30]. These results are consistent with our findings that in the subgroup analysis of sex, the female group showed a stronger association with HL than the male group. 

Inflammation may be another cause of the association between pre-sarcopenic obesity and HL. Obesity itself promotes inflammation by increasing pro-inflammatory cytokines such as interleukin-6 and necrosis factor-alpha [40]. Low muscle mass and obesity are also associated with several inflammatory cytokines, including interleukin-6, serum insulin-like growth factor-1, and hs-CRP [41]. The chronic activation of these inflammatory cytokines could induce HL by infiltrating the endolymphatic sac in the inner ear [42].

In this study, there was a significant association between pre-sarcopenic obesity and HL in women participants. This result is consistent with the results of a previous study, which reported a stronger association between low muscle mass and the prevalence of hearing loss in women more than in men participants [21]. As mentioned in the previous paragraph, women participants are more susceptible to cardiovascular disease and inflammatory disease. Several possible explanations related to the sex-specific differences are that a decline in muscle mass is more pronounced in females [43]. Additionally, women experience an estrogen hormone-deficient state for more than one-third of their lives, which is likely to result in decreased protein synthesis and an increased prevalence of pre-sarcopenia [44]. Therefore, pre-sarcopenic obesity may be more strongly associated with HL in women than in men. 

This study demonstrated that there was a stronger association between pre-sarcopenic obesity and HL in younger subjects compared with elderly subjects. A possible explanation is that inflammation may be a mediator between pre-sarcopenic obesity and HL. The present finding is in line with a previous study, which found a stronger association between high-sensitivity CRP (hs-CRP) and sarcopenic obesity in younger subjects than in the elderly [41]. Even though inflammation is an important patho-mechanism of sarcopenia and obesity in the elderly population, younger subjects can be more susceptible to changes in inflammation [45].

There were higher low-frequency and mid-frequency hearing thresholds in the obesity alone group than in pre-sarcopenia alone group. Furthermore, the prevalence of hearing loss was slightly higher in the obesity group (2.9%) than in the pre-sarcopenia alone group (2.2%). The exact mechanism is not known yet. However, a possible explanation is that the metabolic impact on impairing hearing function can be higher in obesity than pre-sarcopenia. Obesity, which is defined as an accumulation of fat tissues, can have many harmful metabolic effects on multiple organs [46]. On the other hand, sarcopenia is described as a metabolic disease that has adverse effects on many organs. However, in this study, we grouped subjects into a “pre-sarcopenia” group, which was defined by decreased skeletal muscle mass because of the study design outlined in [47]. Therefore, obesity itself may have more deleterious effects on hearing function than pre-sarcopenia alone. Still, further studies comparing sarcopenia versus obesity on hearing loss are needed.

There are some limitations to our study. First, because our study is a cross-sectional study, causal relationships between pre-sarcopenic obesity and HL is difficult to evaluate. Further prospective studies are needed to investigate the causal relationship between them. Second, since we did not consider muscle function due to a lack of information, definite sarcopenia was not considered in the present study. However, there were many clinical studies defining sarcopenia based only on low muscle mass [16,17,48,49], although we determined that low muscle mass defined pre-sarcopenia. Additionally, one’s performance and physical strength decreases consecutively as muscle mass decreases [50], and pre-sarcopenia is a pre-clinical status of sarcopenia-related problems. Third, our study did not consider sensitive components of hearing loss (such as speech discrimination or speech recognition thresholds) because this study is a population-based study with health screening properties. We did not see 265,792 subjects as ENT outpatients but we measured hearing levels during health examinations that were conducted during a medical health check-up. Therefore, we could not check every frequency of 250 Hz, 500 Hz, 1000 Hz, 2000 Hz, 4000 Hz, and 8000 Hz. Because we did not conduct an otoscopic evaluation for all subjects, we could not determine whether the hearing loss was sensorineural or conductive hearing loss. Further studies including evaluations of frequency from 250 Hz to 8000 Hz are needed to determine the association between the type of hearing loss and sarcopenic obesity.

## 5. Conclusions

In this large population study, an increased risk of hearing loss was found for those with a co-existence of low skeletal muscle mass and obesity, even if they were comparatively middle-aged participants. Furthermore, age and sex differences in these associations were found, showing a strong association in younger (<60 years) and women participants than in older and men participants. This study suggests a possible connection between sarcopenic obesity and the development of hearing loss. Future research using randomized trials is needed to show that targeted treatments for sarcopenic obesity can improve hearing function.

## Figures and Tables

**Table 1:** Baseline characteristics of study population (*n =* 265,792).

	Total	Control	Pre-Sarcopenia Alone	Obesity Alone	Pre-Sarcopenic Obesity	*p* Value
Age (years)	42.3 ± 9.4	42.1 ± 8.9	40.4 ± 10.1	43.2 ± 9.8	46.0 ± 13.1	<0.001 *
Men (%)	54.6	54.0	16.2	69.4	35.0	<0.001 ^†^
Screening center (Seoul, %)	60.4	59.6	63.6	60.7	59.8	<0.001 ^†^
Height (cm)	167.5 ± 8.6	168.3 ± 8.2	160.4 ± 6.2	168.8 ± 8.4	159.9 ± 7.5	<0.001 *
Weight (kg)	67.2 ± 13.5	64.8 ± 10.4	49.4 ± 4.9	77.9 ± 12.3	57.2 ± 5.7	<0.001 *
BMI (kg/m^2^)	23.8 ± 3.5	22.7 ± 2.3	19.2 ± 1.4	27.2 ± 3.0	22.4 ± 1.4	<0.001 *
Appendicular skeletal muscle mass (kg)	20.6 ± 5.0	20.8 ± 4.6	14.6 ± 2.4	22.4 ± 4.5	15.2 ± 3.0	<0.001 *
SMI (kg/m^2^)	7.2 ± 1.1	7.3 ± 1.0	5.6 ± 0.5	7.8 ± 0.9	5.9 ± 0.6	<0.001 *
Smoking status (current smoker, %)	16.2	15.80	6.50	20.50	9.70	<0.001 ^†^
Heavy drinker (%)	20.1	19.5	9.5	25.1	12.9	<0.001 ^†^
Regular physical activity (%)	14.9	16.90	9.60	13.10	9.60	<0.001 ^†^
Hypertension (%)	10.4	8.2	4.1	16.3	12.1	<0.001 ^†^
Diabetes mellitus (%)	3.5	2.9	1.9	5.1	4.9	<0.001 ^†^
Dyslipidemia (%)	16.4	14.5	9.0	22.4	18.6	<0.001 ^†^
Fasting glucose (mg/dL)	97.9 ± 16.3	96.4 ± 13.7	92.8 ± 13.6	102.5 ± 19.9	98.2 ± 19.2	<0.001 *
HbA1c (%)	5.5 ± 0.6	5.5 ± 0.5	5.4 ± 0.5	5.7 ± 0.7	5.6 ± 0.7	<0.001 *
Trigrlycerides (mg/dL)	119.1 ± 84.2	107.8 ± 74.8	83.1 ± 43.9	152.6 ± 99.8	112.3 ± 69.8	<0.001 *
Total cholesterol (mg/dL)	191.4 ± 34.4	188.7 ± 32.9	186.0 ± 32.4	197.9 ± 36.4	198.2 ± 37.4	<0.001 *
LDL-C (mg/dL)	126.8 ± 33.2	124.0 ± 32.0	116.2 ± 30.9	135.4 ± 34.3	132.3 ± 35.7	<0.001 *
HDL-C (mg/dL)	59.7 ± 16.1	61.7 ± 16.0	70.5 ± 16.0	52.6 ± 13.1	61.9 ± 15.3	<0.001 *
AST (IU/L)	22.7 ± 14.7	21.4 ± 13.7	19.6 ± 13.4	26.2 ± 16.4	22.3 ± 11.6	<0.001 *
ALT (IU/L)	24.7 ± 21.3	21.3 ± 16.7	16.0 ± 13.6	34.0 ± 27.3	22.0 ± 16.3	<0.001 *

Data are presented as means ± standard deviation, or percentage. *p* values for between group difference by * one-way ANOVA in continuous variables or by ^†^ chi-square test in categorical variables. SMI (kg/m^2^) = appendicular skeletal muscle mass (kg)/height (m^2^). Abbreviations: ALT, alanine aminotransferase; AST, aspartate aminotransferase; BMI, body mass index; CRP, C-reactive protein; HbA1c, glycated hemoglobin; HDL-C, high-density lipoprotein cholesterol; LDL-C, low-density lipoprotein cholesterol; SMI, skeletal muscle mass index.

**Table 2:** Prevalence of hearing loss for the subjects classified by presence of pre-sarcopenia and obesity (*n =* 265,792).

	Control	Pre-Sarcopenia Alone	Obesity Alone	Pre-Sarcopenic Obesity	*p* Value
Classification according to hearing loss					<0.0001
Normal (%)	98.2	97.5	97.0	93.8	
Hearing loss (%)	1.7	2.2	2.9	6.2	

**Table 3:** Multivariable-adjusted odds ratios (95% CI) for prevalence of hearing loss in pre-sarcopenia, obesity, and pre-sarcopenic obesity.

	Control ^1^	Pre-Sarcopenia Alone	Obesity Alone	Pre-Sarcopenic Obesity	*p* Value
Model 1	1.0 (Ref)	1.27 (1.15–1.39)	1.70 (1.61–1.80)	3.77 (3.34–4.25)	<0.0001
Model 2	1.0 (Ref)	1.15 (1.02–1.29)	1.27 (1.19–1.36)	1.33 (1.14–1.57)	<0.0001
Model 3	1.0 (Ref)	1.19 (1.06–1.34)	1.20 (1.12–1.28)	1.30 (1.10–1.56)	<0.0001

^1^ Control = non-pre-sarcopenia and non-obesity. Model 1: crude analysis. Model 2: age, sex, screening center, current smoker, heavy drinker, and regular physical activity. Model 3: Model 2 + hypertension, HbA1c, and HDL-C. Abbreviations: CI, confidence interval; HbA1c, glycated hemoglobin; HDL-C, high-density lipoprotein cholesterol.

**Table 4:** Subgroup analysis for prevalence of hearing loss in pre-sarcopenia alone, obesity alone, and pre-sarcopenic obesity.

	Control ^1^	Pre-Sarcopenia Alone	Obesity Alone	Pre-Sarcopenic Obesity	*p* Value
Sex					
Women (*n =* 120,552)	1.0 (Ref)	1.23 (1.03–1.41)	1.29 (1.14–1.45)	1.44 (1.16–1.80)	<0.0001
Men (*n =* 145,240)	1.0 (Ref)	1.21 (1.00–1.46)	1.15 (1.06–1.25)	1.15 (0.90–1.47)	
Age (years)					
<60 (*n =* 253,149)	1.0 (Ref)	1.17 (1.01–1.38)	1.24 (1.14–1.35)	1.43 (1.11–1.84)	<0.0001
≥60 (*n =* 12,643)	1.0 (Ref)	1.17 (0.98–1.40)	1.12 (0.99–1.26)	1.18 (0.95–1.46)	

^1^ Control = non-pre-sarcopenia and non-obesity. Multivariable-adjusted odds ratios (95% CI) were estimated after adjustments for age, sex, screening center, current smoker, heavy drinker, and regular physical activity, history of hypertension, HbA1c, and HDL-C. Abbreviations: CI, confidence interval; HbA1c, glycosylated hemoglobin; HDL-C, high-density lipoprotein.

## Data Availability

Data can be obtained from the corresponding author upon reasonable request.
